# Mechanical Thrombectomy in Africa: A Challenging Future

**DOI:** 10.7759/cureus.76365

**Published:** 2024-12-25

**Authors:** Oreoluwa Morakinyo, Aliu Yakubu, Augustine C Amuta, Eunice O Mejulu, Bede N Nriagu, Godbless Ajenaghughrure

**Affiliations:** 1 Neurology, University of Texas Medical Branch, Galveston, USA; 2 Geriatric Psychiatry, University Hospital Wishaw, Wishaw, GBR; 3 Health and Wellness, Prince George's County Health Department, Upper Marlboro, USA; 4 College of Medicine, Western Illinois University, Macomb, USA; 5 Internal Medicine, New York Medical College, Metropolitan Hospital Center, New York, USA; 6 Internal Medicine, Good Samaritan Hospital, Cincinnati, USA

**Keywords:** africa, endovascular intervention, mechanical thrombectomy (mt), neuroimaging and neurointervention, stroke

## Abstract

Mechanical thrombectomy (MT) has revolutionized the management of proximal large vessel occlusions (LVOs) in patients with acute ischemic stroke (AIS), improving long-term outcomes compared to standard treatments. However, despite its success in high-income countries, the widespread implementation of MT in Africa remains limited. With Africa experiencing one of the highest stroke burdens globally, this study examines the barriers impeding the adoption of MT in the region. These challenges include insufficient healthcare infrastructure, lack of specialized training, and economic constraints, which hinder timely access to care. The review highlights significant disparities in stroke management across African countries and proposes strategies to enhance access to stroke care, emphasizing the need for policy reforms, infrastructure investment, and specialized training to improve outcomes for African stroke patients.

## Introduction and background

In the last decade, mechanical thrombectomy (MT) has gained global prominence and acceptability in the management of stroke, significantly transforming the treatment of patients with large vessel occlusions (LVOs) [1]. Some studies have shown that between 5-13% of patients with ischemic stroke present in the recommended intra-arterial treatment window of three to six hours [2]. Demeestere et al. reported that approximately 10-15% of eligible stroke patients receive intravenous thrombolysis (IVT), and endovascular thrombectomy is administered to about 8-10% of patients with ischemic stroke, reflecting both expanded treatment windows and advances in intervention strategies [3]. 

MT is cost-effective and significantly improves patients’ post-stroke functional outcomes. In a study by van den Berg et al., the mean costs per patient who had an interventional neurology procedure were over $126,000 compared to $143,000 for those without interventional neurology procedures [4]. Aronsson et al. observed that combining thrombectomy with stent retrievers in guideline-based care (including IVT) resulted in a notable gain of 0.40 life years and 0.99 quality-adjusted life years, along with a cost savings of approximately $221 per patient [5]. 

Despite these benefits, racial and socioeconomic disparities exist in the accessibility and utilization of MT for acute stroke. While MT was widely used in the United States, African American and low-income patients were less likely to receive it [6]. Access to MT is limited in many low- and middle-income countries, with an access rate of 0.48% compared to 21.56% in high-income countries. Notably, Among African countries, Egypt has the highest access to MT at 2.8%, followed by South Africa at 2.5%, Sudan at 2.1%, Tunisia at 1.1%, and Kenya at 0.4% [7]. 

Stroke is a leading cause of death and disability in Africa, with the burden increasing due to aging populations and a rise in risk factors such as hypertension and diabetes [8]. However, the infrastructure to manage acute strokes, including access to MT, is often lacking. This paper aims to explore the current state of MT in Africa, highlighting the challenges in its implementation and proposing strategies for improving access and outcomes.

## Review

Present challenges of mechanical thrombectomy in Africa

Acute Stroke Care Centers and Finances

A stroke care center is designated with a multidisciplinary team specialized in stroke management. Despite the available evidence of the effectiveness of these centers, they often lack comprehensive services and adherence to best practices. For instance, a study in Nigeria showed that only five out of 58 hospitals have dedicated stroke units, with limited bed spaces ranging from 10 to 27, resulting in a lack of bed spaces for all stroke patients [9]. Similarly, only 9.1% of tertiary hospitals in Ghana had a dedicated stroke unit, leaving the majority of stroke patients to be treated on standard medical wards with no access to IVT and MT services [10]. Stroke management is usually provided in the emergency department with limited access to thrombolysis [8]. A cross-sectional study of 17 African countries showed that only five countries (Algeria, Egypt, Tunisia, Nigeria, and Morocco) had established dedicated stroke units and stroke centers limited to Egypt and Algeria [11]. Moreover, MT services are available in just six countries (Egypt, Nigeria, Algeria, Morocco, Kenya, and South Africa), with procedure rates below 5%. This highlights the need for more investment in the health sectors and enhanced stroke awareness programs in order to reduce the high fatality and disability rate associated with stroke in Africa.

In a hospital survey in Ghana, only 36.4% and 27.3% had neurologists and neurosurgeons as part of their stroke team, with no occupational therapists or speech therapists [10]. The World Health Organization (WHO) recommends at least one neurologist per 100,000 people, yet the WHO African region has a median of only 0.043 neurologists per 100,000 people, primarily concentrated in urban areas [12]. Addressing this shortage of well-trained specialists is crucial to ensuring comprehensive stroke management, facilitating the effective operation of stroke units, and enhancing thrombectomy services. 

Government involvement in healthcare financing has been identified as the global primary driver of healthcare funding. Providing neurological interventional services such as MT is costly for patients and the government. In the United Kingdom, the average cost of providing MT within the first 72 hours was £12,440 [13]. The cost of care for stroke patients in Africa varies depending on the setting, ranging from 600 USD to 4860 USD [14]. In Tanzania, an average of 220 USD is spent on stroke patients [15]. Ghana lacks government support for stroke services, including stroke rehabilitation and community awareness [9]. This difference underscores the substantial disparity in health financing between high-income countries and African nations. In 2009, the High-Level Task Force on Innovative International Financing for Health Systems recommended that low-income countries should allocate a minimum of US$ 44 per capita on health; 22 out of the 45 African countries spending less than the recommendation [16].

Emergency Services Access

Studies have shown that patients with stroke present to the emergency room beyond the recommended window period of six to 16 hours for MT in cases of LVOs [17] due to limited access to emergency services needed for early presentation, and this remains a significant healthcare concern in Africa. According to Ogbole et al., no patient presented for imaging within three hours of ictus, and only 31% presented within 12 hours, with 46% failing to present within 24 hours [18]. Another study in Burkina Faso found that the average time from door to computer tomography scan was 22 hours [19]. Barriers to early presentation include traffic congestion, absence of designated ambulance lanes, dependence on privately owned vehicles for transportation, and limited public awareness of stroke signs and symptoms [20-22]. Though some highly developed emergency services, such as helicopter medical services, have been developed, the economic difficulties faced by most African countries prevent easy access to these services for the general public. According to a survey of emergency services in 49 African countries, 33 lack evidence of emergency services and access to emergency telephone services [23].

Additionally, except in South Africa, emergency services are underutilized, with most countries conducting fewer than 10,000 transports annually. The response time of emergency services in Africa exceeds the WHO's recommended time of eight minutes [24]. A study of emergency medical service (EMS) in Africa found that response time varies by location, with a mean response time of 24 minutes in urban settings and rural areas having considerably longer mean response time of 109 minutes [23]. A study conducted in Ghana reported an average response time of 16.9+/-0.7 minutes and a median transport time of 82 minutes, ranging from five to 552 minutes [20].

Imaging Studies

Over the past four decades, the MT procedure was developed utilizing imaging techniques to guide catheters and other endovascular instruments into the arterial circulation. The accessibility of imaging services in Africa remains limited. A study among West African countries showed that some countries lack access to MRI scanners, and Nigeria accounts for two-thirds of the available MRIs in the region [25]. A survey in Ghana found that only 63.6% of the surveyed hospitals had functional CT scans, while the availability of MRI and Doppler ultrasonography was lower at 27% and 21%, respectively, with only a few offering 24-hour service [9]. When available, the cost of these services is often very high, thereby preventing patients with LVOs from receiving optimal care based on evidence-based practices.

Future of mechanical thrombectomy in Africa

Stroke remains a leading cause of morbidity and mortality globally, with developing countries accounting for approximately 85% of global stroke-related deaths [26]. In Africa, the future of MT lies in efforts to address barriers such as limited public awareness of stroke symptoms, inadequate healthcare infrastructure, and insufficient resources for stroke care.

Addressing public awareness and education

One critical barrier to stroke management in Africa is the limited awareness of stroke symptoms, which often delays presentation and treatment. Cultural and religious beliefs significantly influence health information dissemination across African communities, as highlighted by Akinyemi et al., who emphasized the role of these factors in the low awareness of stroke signs and symptoms [8]. Engaging traditional and religious leaders could significantly enhance stroke awareness and dispel cultural misconceptions about stroke. Health campaigns targeting schools present another effective avenue for promoting stroke awareness. Studies indicate that educating adolescents about stroke symptoms can enhance their knowledge of stroke signs and symptoms [27]. Non-governmental organizations also play a pivotal role in public education; in Nigeria, groups such as Stroke Care International and Acha Memorial Foundation have made significant strides through community campaigns in marketplaces, social media, and the release of bimonthly magazines [28].

Enhancing emergency medical services and infrastructure

Timely access to medical care is crucial for effective MT, but logistical challenges like poor road networks, traffic congestion, and the absence of designated ambulance lanes prevent timely patient presentation. Nigeria, despite establishing its first emergency medical services (EMS) in 2001, has struggled to develop a comprehensive nationwide service due to factors like inadequate infrastructure and trained personnel [29]; for MT to thrive, the government should make roads to hospitals accessible by developing emergency lanes for ambulance services, expanded air travel options for patient transport, and equipping ambulances with necessary resuscitation equipment. The private sector can also support the government by donating ambulance vehicles to primary, secondary, and tertiary centers to facilitate prompt response to care, and continuous training for EMS staff could also enhance response times and improve outcomes.

Expanding stroke care centers and mechanical thrombectomy accessibility

The availability of MT services in Africa remains limited but is gradually expanding. For example, a study in Egypt demonstrated that government investment in stroke care, including funding for alteplase and training of stroke teams, significantly increased intravenous thrombolysis eligibility rates [30]. This underscores the importance of government investment in establishing and maintaining MT centers and training healthcare providers. However, in many African countries like Nigeria, there is a heavy reliance on government funding, which remains inadequate. The budgetary allocation to healthcare remains a small fraction of overall public spending, leaving many regions without sufficient MT centers. The government needs to expand insurance coverage to more people. In Kenya, the population has been sensitized and enrolled in the National Health Insurance Fund as a pathway to access universal healthcare and to reduce healthcare expenditure incurred by patients, including stroke patients in the country [31]. There is a need to collaborate with the private sector to establish new acute stroke centers and improve existing centers; there also needs to be a modality for maintaining both new and existing centers. Establishing smaller stroke centers across sub-Saharan Africa may help reduce mortality from stroke, considering that sub-Saharan Africa has the highest prevalence of stroke. A study from Guinea established that minimally equipped stroke units significantly reduced stroke mortality [32].

Improving access to imaging services

Effective MT requires advanced imaging techniques such as computed tomography (CT) and magnetic resonance imaging (MRI). However, access to these services in Africa remains limited due to high costs and poor maintenance infrastructure. Many healthcare facilities lack functional imaging equipment, and maintenance and repair challenges hinder service delivery when available. Addressing this issue requires government investment in providing and maintaining imaging equipment and support from private entities, such as equipment donations. Kenya’s Managed Equipment Services initiative is a potential model for improving imaging access, as it has equipped numerous national and county hospitals with CT scans [31]. Similar efforts across Africa could enhance diagnostic capabilities, thus improving the quality and reach of MT services.

Emphasis on primary prevention and training

A sustainable future for MT in Africa also requires focusing on primary stroke prevention and strengthening the healthcare workforce. Improving epidemiological surveillance, routine risk factor assessments, and health education can help reduce the incidence of stroke [8,22]. Investments in acute stroke care planning and workforce development should complement primary prevention efforts, including specialized training in stroke management for physicians, nurses, and allied health professionals. Including stroke medicine in postgraduate curricula and offering targeted short courses could help address the shortage of qualified stroke professionals [8].

Moreover, initiatives like the African Stroke Control Observatory Risk Reduction Ecosystem (A-SCORE), which operates under the World Stroke Organization and WHO, aim to coordinate stroke prevention and management efforts across the continent [33]. Such programs could address conventional and novel barriers to stroke care, enhancing the overall quality of stroke management in Africa.

Role of the Society of Vascular and Interventional Neurology

The Society of Vascular and Interventional Neurology (SVIN) is an international society composed of specialists and subspecialists involved in the interventional management of cerebrovascular and neurological diseases. Its mission is to advance interventional neurology as a field, with the ultimate goal of improving the clinical care and outcomes of patients with stroke and cerebrovascular diseases [34]. For Africa to have a future in interventional neurology, the SVIN has a significant role in rallying the few interventional neurologists in Africa and involving them in all collaborative initiatives. MT2020+ and ALLM partnership is an initiative seeking to reduce the barrier and improve access to MT by utilizing public health tools, electronic medical record applications, and health intervention to double worldwide access to MT [34]. Trainees should be encouraged to attend conferences and collaborate on publications and research. Thus, there is a need for more collaboration, particularly between interventional neurologists in Africa and the rest of the world.

## Conclusions

The future of MT in Africa depends on a multifaceted approach that addresses public awareness, infrastructure, healthcare financing, and professional training. While significant challenges remain, including economic barriers and inadequate healthcare infrastructure, targeted investments and collaborations between governments, the private sector, and international organizations can foster an environment where MT becomes more accessible and effective across Africa. By addressing these systemic challenges, Africa can enhance its capacity to provide life-saving MT services, ultimately improving outcomes for stroke patients and reducing the region’s high stroke burden.
